# A cardiovascular-motor axis framework for perfusion-mediated motor impairment

**DOI:** 10.1016/j.ijcha.2026.101995

**Published:** 2026-08-22

**Authors:** Matteo Scorcelletti, Armin Weers, Bastian Schrader, Joachim Schrader, Alessandro Del Vecchio, Albrecht Elsässer

**Affiliations:** a University Clinic for Internal Medicine – Cardiology, Department of Human Medicine, Carl von Ossietzky Universität Oldenburg, Klinikum Oldenburg, Rahel-Straus-Straße 10, 26133 Oldenburg, Germany; b Institute for Hypertension and Cardiovascular Research (INFO), Soestenstraße 18, 49661 Cloppenburg, Germany.; c Department of Artificial Intelligence in Biomedical Engineering, Friedrich-Alexander University Erlangen-Nürnberg, Henkestraße 91, 91052 Erlangen, Germany.

**Keywords:** Cerebral hypoperfusion, Heart failure, Motor impairment, Peripheral arterial disease, Cardiovascular-motor axis

## Abstract

Cardiovascular disease is the leading cause of death worldwide, and among survivors of heart failure, peripheral arterial disease, valvular disease, and atrial fibrillation many develop motor impairment: weakness, slow gait, sarcopenia, frailty. These deficits are usually attributed to deconditioning or coexisting conditions. This narrative review advances a different hypothesis: that reduced arterial blood delivery, from a failing pump or a narrowed artery, itself drives motor impairment at every level of the motor pathway, from cortex to muscle fibre. We group cardiovascular conditions into systemic models of low blood flow (heart failure with reduced or preserved ejection fraction, low-flow aortic stenosis, atrial fibrillation) and regional models of arterial obstruction (carotid, subclavian, and vertebral stenosis; peripheral arterial disease), quantifying the perfusion deficit where haemodynamic data exist. We then examine mechanisms: cerebral hypoperfusion that thins the motor cortex and weakens motor planning; vulnerability of the cerebellum, basal ganglia, and spinal motor neurons, impairing coordination and gait; ischaemic peripheral nerve damage that denervates muscle; loss of muscle capillaries, mitochondrial injury, and failed regeneration; amplified feedback from underperfused muscle that suppresses central motor drive; and gating of corticospinal excitability by the cardiac cycle. Causality is supported by within-patient comparisons, in which a lateralised ischaemic limb or hypoperfused hemisphere weakens while the opposite side, under identical systemic conditions, does not; by graded perfusion-to-outcome relationships; and by recovery after cardiac transplantation, ventricular assist device support, valve replacement, and revascularisation. Deconditioning amplifies these effects rather than causing them, so treatment should restore blood flow and rebuild muscle together.

## 1 Introduction

Cardiovascular disease remains the leading cause of death worldwide, accounting for approximately 18.6 million deaths and 523 million prevalent cases in 2019 [1]. Mortality statistics, however, capture only part of its burden. Among the millions who survive cardiovascular diseases such as heart failure and peripheral arterial disease (PAD), a substantial proportion experience a decline in physical function that extends well beyond the cardiac impairment itself, with effects on motor performance and quality of life [2], [3], [4].

In heart failure, quadriceps and leg strength are up to 26% lower than in age-matched controls [5], [6], peak oxygen consumption is reduced by approximately 40% [5], and maximal inspiratory pressure is reduced by 24% [7], with 30–50% meeting criteria for inspiratory muscle weakness [8]. In peripheral arterial disease (PAD), affecting approximately 230 million worldwide [4], six-minute walk distance is reduced by approximately 21% [9], with continued decline even when leg symptoms appear stable [4]. In severe aortic stenosis, six-minute walk distance before transcatheter aortic valve implantation (TAVI) averages only 204 m, approximately half the expected value [10], [11]; without intervention, mortality approaches 38% at one year and 68% at five years [12]. Grip strength tracks disease severity, with each kilogram of additional strength associated with a 2% lower risk of a worse New York Heart Association class, and 29% of patients meeting criteria for low grip strength [2], [13]. This gradient should be read cautiously: New York Heart Association class reflects symptom burden rather than cardiac impairment alone, and the frailty and sarcopenia that lower grip strength also worsen functional class, so the association is consistent with, but not proof of, a perfusion effect.

Sarcopenia affects approximately 34% of heart failure patients overall and up to 55% of those hospitalised [14], with a pooled prevalence across cardiovascular disease of 35% versus 13% in the general population [15]. At least half of all heart failure patients meet criteria for frailty, reaching up to 90% in heart failure with preserved ejection fraction (HFpEF) versus 30–60% in heart failure with reduced ejection fraction (HFrEF), and approximately 26% higher in women than men [16]; the higher HFpEF prevalence reflects the older age and greater comorbidity burden in this population, with greater than 60% having at least four comorbidities [17].

Physical functional performance on standardised tests independently predicts hospitalisation and mortality in heart failure [18], yet the neuromuscular axis in cardiovascular disease remains poorly characterised in clinical practice. Cardiac rehabilitation focuses on aerobic reconditioning, with resistance training and lean mass assessment underutilised [3]. Finer-grained measurements that would detect neural-level pathology, motor unit recruitment, voluntary activation, and regional tissue oxygenation during effort, are rarely performed in cardiovascular populations. Whether the observed motor impairment reflects disuse alone or a direct consequence of reduced perfusion cannot be resolved without such data.

Although these deficits are commonly attributed to deconditioning or to comorbid conditions such as ageing, diabetes, or frailty [2], accumulating evidence suggests this explanation is incomplete, with the most revealing observations coming from conditions that produce asymmetric or lateralised deficits. In aortic dissection with limb malperfusion, the affected limb loses motor function while the contralateral limb, receiving normal arterial supply, remains intact. This within-patient asymmetry cannot be explained by deconditioning, medication effects, or systemic inflammation [19]. Similarly, subclavian artery stenosis, present in 2 to 4% of the general population and up to 18% in patients with peripheral arterial disease [20], produces unilateral upper limb claudication in up to 57% of affected patients [21], with isokinetic dynamometry confirming 42% less work capacity in the ischaemic arm compared to the contralateral side within the same patient [22]. Carotid stenosis produces lateralised cortical thinning and perfusion deficits in the motor cortex ipsilateral to the stenosis [23], accompanied by cognitive [24] and motor function impairment [25]. These natural within-subject experiments provide the most direct evidence that reduced arterial blood flow, not deconditioning or systemic disease, drives the observed motor impairment.

A dose-response gradient further supports a perfusion-mediated mechanism. In heart failure, functional capacity broadly declines across New York Heart Association classes I through IV, paralleling the progressive reduction in cardiac reserve [2]. The prevalence of sarcopenia mirrors disease severity: 30% in cardiac arrhythmia, 32% in chronic heart failure, and 43% in coronary artery disease [15]. In PAD, the ankle-brachial index provides a continuous measure of perfusion adequacy, and functional impairment worsens in a graded fashion from mild disease (ankle-brachial index 0.7–0.9) through moderate (0.5–0.7) to critical limb-threatening ischaemia (below 0.4). In aortic stenosis, valve area and stroke volume index correlate with exercise limitation. In each case, worse blood flow predicts worse motor function.

**Fig. 1:**
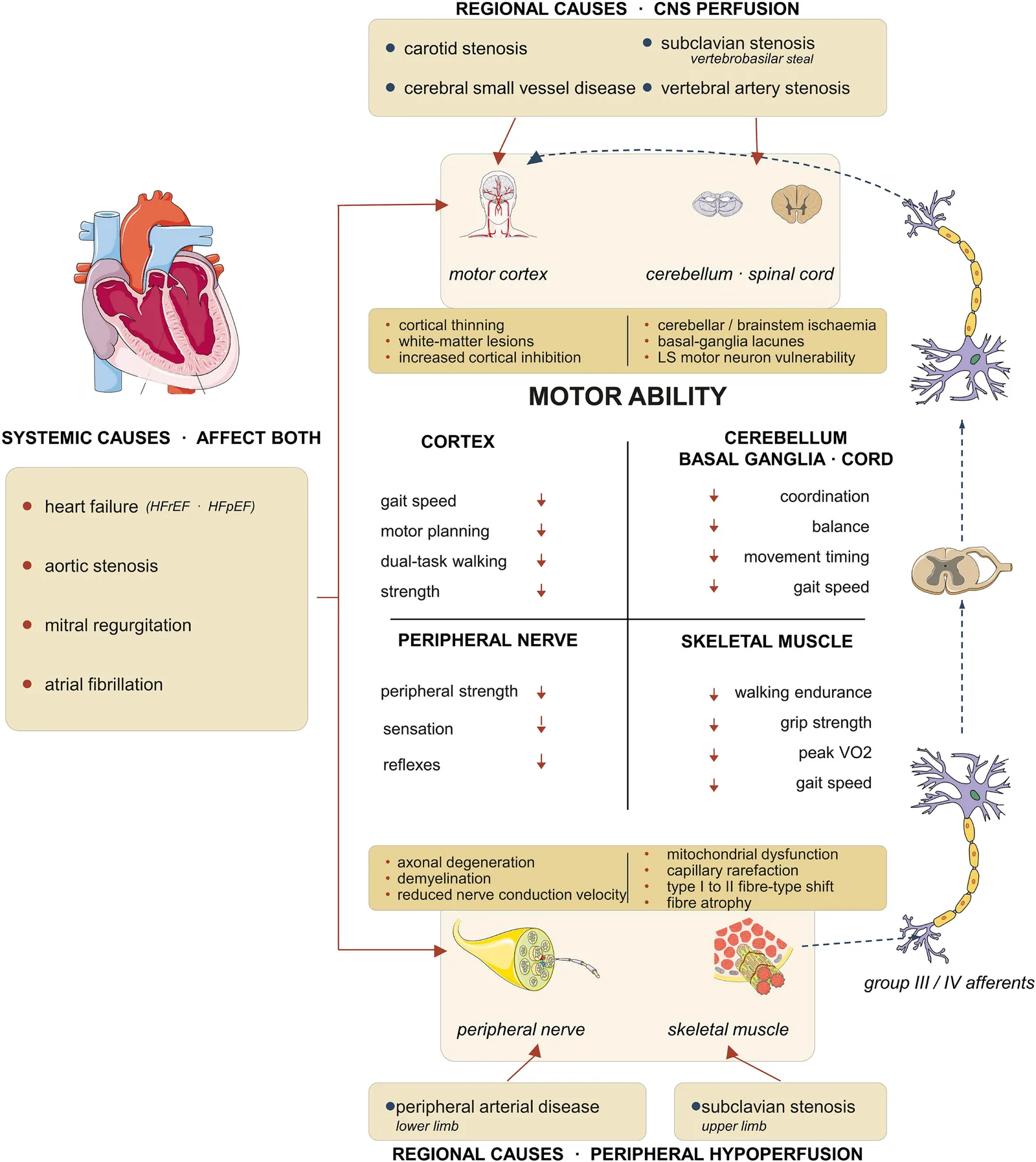
The cardiovascular-motor axis framework. Concentric reading: outer ring, cardiovascular causes; inner ring, affected tissue; yellow band, tissue-level pathology; centre, motor consequence. Causes are grouped by territory: regional CNS perfusion (top), regional peripheral hypoperfusion (bottom), and systemic low-output (left). Abilities listed across more than one tissue quadrant (gait speed, walking endurance) reflect convergent multi-pathway contributions. Cerebral small vessel disease represents cumulative exposure rather than discrete events. The neuron at right (with axon and terminal projecting toward motor cortex) depicts the group III/IV afferent feedback loop from skeletal muscle to the central nervous system. HFrEF, heart failure with reduced ejection fraction; HFpEF, heart failure with preserved ejection fraction; BG, basal ganglia. Tissue icons: Servier Medical Art (CC BY 4.0). Neuron icon: derived from Wikimedia Commons (CC BY-SA 3.0).

This review examines the hypothesis that reduced arterial blood delivery, whether caused by a failing cardiac pump or by obstruction of specific arterial territories, is itself a driver of motor impairment operating through mechanisms that span the full length of the neuromuscular axis from the motor cortex to the muscle fibre (Fig. 1). The scope is restricted to conditions where the primary pathophysiology is mechanical: the heart ejects less blood per minute, or a specific artery is narrowed or occluded. Conditions in which perfusion deficits arise from primary microvascular pathology (such as diabetic microangiopathy, sepsis-related microcirculatory failure, or hypertensive microvascular remodelling) are excluded, because in those settings the blood flow variable cannot be isolated from confounding tissue-level pathology. PAD, by contrast, is included as a macrovascular condition: although it produces secondary microvascular changes, the primary lesion is large-artery atherosclerotic obstruction.

**Scope and approach.** This is a narrative review. Because the question spans a range of conditions and the whole neuromuscular axis, and the outcomes at each level are not on a common scale, there is no single question to pool and no summary effect estimate would be meaningful.

**Fig. 2:**
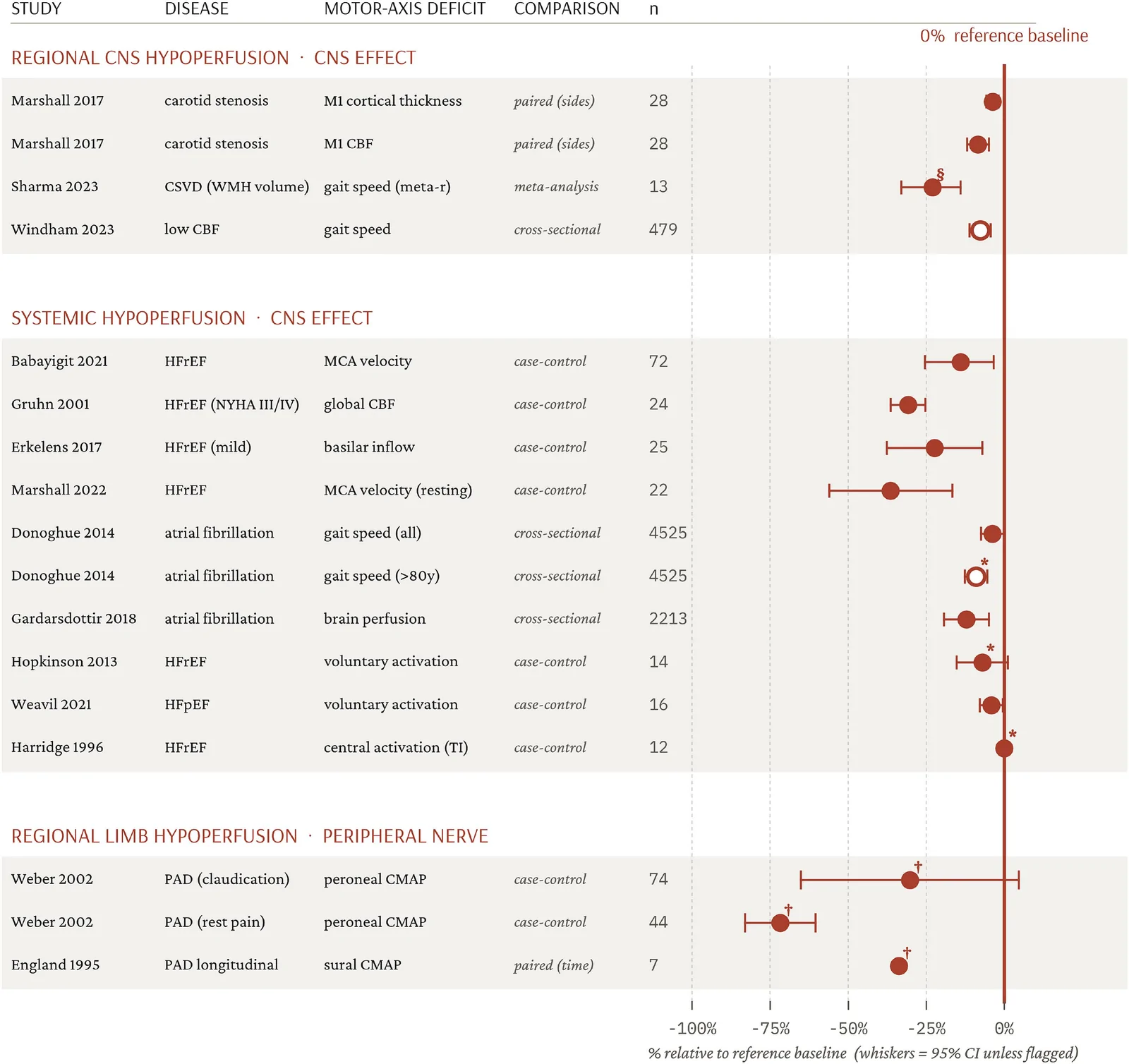
Quantifiable deficits across the cardiovascular-motor axis: nervous system. Forest plot of case-versus-reference deficits, grouped to mirror Fig. 1‘s three perfusion routes: regional CNS hypoperfusion, systemic hypoperfusion, and regional limb hypoperfusion (peripheral nerve). Each marker is the % deficit reported by the cited paper; whiskers show 95% confidence intervals unless flagged. Open markers are regression-model predictions. The comparison column gives the design of the plotted comparison rather than of the paper, so a study contributing two rows can carry two labels. For meta-analysis rows, n is the number of contributing studies. † within-group interquartile range or no inferential statistic on the mean; * interval approximated from a published *p*-value bound or paper-reported null; § meta-analytic correlation coefficient plotted as r × 100. The reference baseline varies by row (healthy controls, the unaffected hemisphere or limb of the same patient, or a stated non-healthy comparator as labelled); Sharma 2023 (§) is a meta-analytic correlation and Windham 2023 a regression-slope projection rather than a between-group contrast. Cross-row magnitudes are not strictly equivalent; the % axis unifies magnitudes visually rather than on a single clinical scale. CBF, cerebral blood flow; CMAP, compound muscle action potential; CSVD, cerebral small-vessel disease; HFpEF, heart failure with preserved ejection fraction; HFrEF, heart failure with reduced ejection fraction; M1, primary motor cortex; MCA, middle cerebral artery; NSVT, non-sustained ventricular tachycardia; NYHA, New York Heart Association; PAD, peripheral artery disease; TI, twitch interpolation; WMH, white-matter hyperintensity.

**Fig. 3:**
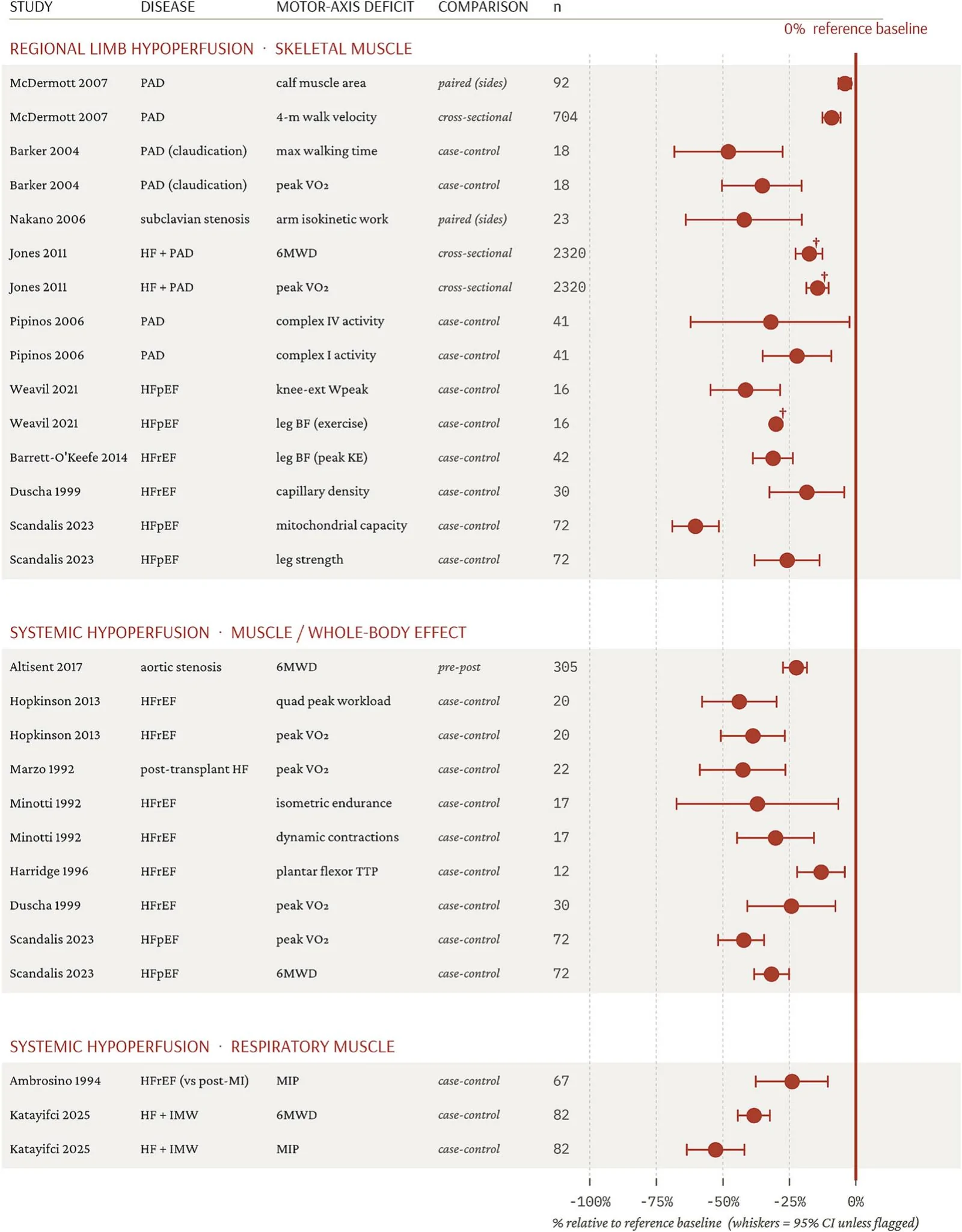
Quantifiable deficits across the cardiovascular-motor axis: skeletal muscle. Forest plot of case-versus-reference deficits, grouped by perfusion locus: regional limb hypoperfusion with muscle outcome, systemic hypoperfusion with muscle or whole-body outcome, and systemic hypoperfusion with respiratory-muscle outcome. Marker, whisker and comparison conventions as in Fig. 2. The reference baseline is healthy controls except where stated otherwise in the disease column (post-MI patients for Ambrosino 1994, HF without PAD for Jones 2011). Cross-row magnitudes are not strictly equivalent because they compare different outcome types (limb blood flow, mitochondrial enzyme activity, capillary density, peak oxygen uptake, walking distance, inspiratory muscle strength); the axis unifies magnitudes visually rather than on a single clinical scale. Three papers (Hopkinson 2013, Weavil 2021, Harridge 1996) also contribute rows to Fig. 2. 6MWD, six-minute walk distance; BF, blood flow; HF, heart failure; HFpEF, heart failure with preserved ejection fraction; HFrEF, heart failure with reduced ejection fraction; IMW, inspiratory muscle weakness; KE, knee extension; MI, myocardial infarction; MIP, maximum inspiratory pressure; PAD, peripheral artery disease; TTP, twitch time-to-peak; VO₂, peak oxygen uptake; Wpeak, peak work rate.

We searched PubMed and Google Scholar up to early 2026 for each condition considered here, combining perfusion terms (cerebral blood flow, ankle-brachial index, cardiac output) with motor outcomes at any level of the axis, from cerebral perfusion and cortical thickness through corticospinal excitability, voluntary activation and nerve conduction to grip strength, gait speed and walking distance, so that each deficit could be placed on the axis rather than read only as a functional endpoint (Fig. 2, Fig. 3). We prioritised studies reporting both measures with perfusion isolable, favouring within-patient asymmetry, dose-response and pre-post designs, and took each comparison against age-matched controls, the unaffected side, or the patient's own baseline, holding age constant. Diabetes was handled study by study: the tissue-level PAD evidence comes from cohorts that excluded diabetic participants [26], [27], while the heart failure and community cohorts adjust for it [28]. Figure percentages are the difference from the stated reference, as a percentage of that reference, with correlation coefficients and regression slopes flagged as such. The argument runs in three steps: conditions are grouped as systemic (reduced pump output) or regional (arterial obstruction) models of low flow, with the deficit quantified where haemodynamic data exist; mechanisms are traced along the axis from cortex to muscle fibre; and causality is weighed against within-patient asymmetry, dose-response gradients, and reversibility after transplantation, valve replacement, endarterectomy and revascularisation.

## 2 Mechanisms: how reduced blood flow impairs motor function

The cardiovascular conditions considered here reduce arterial blood delivery either systemically (failing pump output) or regionally (arterial obstruction). Systemic models include heart failure with reduced ejection fraction (HFrEF), which cuts resting cardiac index by up to 40% in severe disease [29] and lowers perfusion globally; HFpEF and severe mitral regurgitation, which preserve resting output but leave forward cardiac output insufficient under effort demand [10], the limit unmasked mainly during exercise and compounded at the tissue level by microvascular and mitochondrial dysfunction [30], [31]; heart failure with mildly reduced ejection fraction (HFmrEF), an intermediate phenotype for which perfusion-motor data are sparse and largely extrapolated from the reduced and preserved groups; severe aortic stenosis, where the low-flow, low-gradient phenotype reduces forward stroke volume [10]; and atrial fibrillation, which removes the 15–25% atrial contribution to ventricular filling and introduces stroke-volume variability. Regional models include carotid stenosis, which reduces ipsilateral cerebral perfusion [23]; PAD, which produces graded limb ischaemia indexed by the ankle-brachial index [4], [32]; and subclavian stenosis, which compromises the ipsilateral upper limb and, via reversed vertebral flow, the posterior cerebral circulation [33]. Heart failure with concurrent PAD layers systemic underperfusion onto a regionally compromised territory [34], [35]. These groups fall along the four domains of cardiovascular ageing described by Troy et al. [36]: vascular, myocardial, valvular, and electrophysiologic. That account addresses why these conditions arise; this review asks what they do to motor output.

PAD evidence discussed here is from atherosclerotic disease: transcriptomic biopsy studies excluded diabetic participants [26] and an animal model of isolated arterial occlusion reproduced the full PAD myopathy [37], so arterial ischaemia per se drives the skeletal-muscle pathology. These mechanisms are not mutually exclusive: in most patients several operate simultaneously, on timescales of minutes to years.

### 2.1 Central nervous system hypoperfusion and motor output

The brain receives approximately 15% of resting cardiac output despite constituting only 2% of body mass [38]. The motor cortex, premotor cortex, supplementary motor area, and associated regions require sustained perfusion to maintain neural activity. If blood flow to these regions falls, the capacity to generate and transmit descending motor commands through the corticospinal tract may be compromised, producing weakness even if the muscle and peripheral nerve are intact. The cerebral-perfusion, motor-cortex, gait, and peripheral-nerve deficits documented across the conditions considered here, measured against healthy controls or the unaffected side, are summarised in Fig. 2.

#### 2.1.1 Systemic hypoperfusion: heart failure and aortic stenosis

Cerebral blood flow is reduced by approximately 31% in severe heart failure with reduced ejection fraction [39], with transcranial Doppler studies showing 14% mean middle cerebral artery velocity reductions [40] and review estimates spanning 14–30% across cohorts [41]. Preferential hypoperfusion affects posterior cortical regions including the precuneus, cuneus, and posterior cingulate [42].

Internal carotid artery blood flow was the sole independent predictor of executive function in heart failure patients, outperforming all cardiac parameters including ejection fraction and cardiac output [43]. Cognitive impairment affects up to 43% of heart failure patients, with 2.6-fold higher odds than in age-matched controls [44], and lower baseline cardiac output predicts faster cognitive decline prospectively over 3.5 years of follow-up [45]. Executive function and motor planning share overlapping prefrontal and premotor substrates [46].

In severe aortic stenosis, the low-flow, low-gradient phenotype produces a chronic low-output state [10]. The ventricle must generate supranormal pressures to eject blood past the narrowed valve, leading to concentric hypertrophy and, eventually, systolic dysfunction that further compromises cerebral perfusion.

Despite autoregulation, cardiac output independently modulates cerebral blood flow [47], so chronic low pump output reduces brain perfusion even within the autoregulatory pressure range. The deficit accumulates over years but reverses when cardiac output is restored [39], [47].

#### 2.1.2 Regional hypoperfusion: carotid stenosis

Carotid artery stenosis is the clearest single model of regional cerebral hypoperfusion; moderate asymptomatic stenosis (≥50%) reaches 5–7.5% in those aged 80 and above and severe stenosis (≥70%) affects up to 3.1% of older men [48]. As autoregulatory reserve is exhausted, cerebral blood flow declines, oxygen extraction rises to maintain metabolism, and tissue ischaemia follows when extraction capacity is exceeded [49].

The middle cerebral artery, which supplies the primary motor cortex for the contralateral face, upper limb, and trunk, is a direct distal branch of the internal carotid artery. Significant carotid stenosis reduces cerebral blood flow by approximately 8% in the motor cortex [23] and by up to 18% in ipsilateral watershed areas, with cerebrovascular reactivity impaired by up to 24% [50]. The motor cortex is particularly vulnerable because it lies at the border zone between middle and anterior cerebral artery territories, where perfusion is most sensitive to upstream flow reductions. The cognitive consequences of carotid-mediated hypoperfusion are lateralised: left stenosis preferentially affects verbal function and right stenosis preferentially affects visuospatial function, correlating with hemodynamic severity [51].

#### 2.1.3 Rhythm-dependent hypoperfusion: atrial fibrillation

Atrial fibrillation affects approximately 37.6 million people worldwide and is projected to reach 62.5 million by 2050 [52], with prevalence rising to 10–17% in those aged 80 and above [28], [52]. It reduces total cerebral blood flow by approximately 13% [53], and the beat-to-beat irregularity may be as damaging as the absolute reduction [54]; paroxysmal atrial fibrillation without significant carotid stenosis produces non-embolic hemodynamic disturbances in the middle cerebral arteries even without overt stroke. Atrial fibrillation carries a 39% increased risk of cognitive impairment and 2.7-fold after stroke [55]. Beyond cognition, atrial fibrillation is independently associated with impaired mobility: in a nationally representative community cohort it predicted slower gait speed and Timed Up-and-Go performance after adjustment for age, cardiovascular comorbidity, frailty and medications, the gait deficit being about 3% at age 70 and a projected 10% at age 89, relative to the mean gait speed of participants without atrial fibrillation [28]. Sustained atrial fibrillation is not a form of heart failure, but the loss of atrial contribution and the stroke-volume variability described above make it a chronic low-output-reserve state, converging with heart failure on the same downstream consequences: reduced perfusion reserve, exercise intolerance, and the deconditioning that follows. The irregular rhythm may also disrupt the normal cardiac-phase gating of corticospinal excitability [56], [57], a non-perfusion mechanism distinct from the flow deficit. Catheter ablation reverses the flow deficit: in nonparoxysmal atrial fibrillation with restored sinus rhythm, cerebral blood flow rises by about 13%, while it continues to decline under medical management [58], providing reversibility evidence specific to this arrhythmia. The gain is specific to the sustained form: nonparoxysmal atrial fibrillation independently predicted the rise in flow (odds ratio 7.7), while paroxysmal patients in sinus rhythm showed none, their flow already at the level the sustained group reached only after ablation [58], so the prediction applies to persistent disease rather than to paroxysmal episodes. In heart failure with persistent atrial fibrillation, a randomised comparison of ablation with rate control found peak oxygen consumption 19.9% higher at twelve months (95% CI 3.9 to 35.9%), with no difference at three months [59], a delay that matches the months-long recovery seen after other flow-restoring interventions.

#### 2.1.4 Motor cortex consequences

In asymptomatic unilateral ≥80% carotid stenosis, cortical blood flow and cortical thickness are coupled in the motor cortex bilaterally (*p* < 0.001), with both reduced on the occluded side [23]; primary motor cortex is 3.7% thinner ipsilateral to the occlusion (*p* < 0.001) while primary visual cortex shows no asymmetry [60]. This within-patient dissociation is the clearest evidence that chronic hypoperfusion damages the motor cortex specifically; whether it translates to corticospinal excitability changes remains untested.

At rest in heart failure, central motor drive is not impaired: enhanced fatigue reflects peripheral abnormality rather than descending-command failure [61], [62]. Voluntary activation nonetheless trends lower at baseline, post-exercise cortical depression is attenuated [5], and activation declines more steeply during exercise [30], consistent with a corticospinal system near its floor. In carotid stenosis, prolonged cortical silent period indicates increased cortical inhibition in the chronically hypoperfused brain [63]. Executive function and motor planning also decline with cerebral hypoperfusion [24], [51], impairing tasks requiring planning, adaptation, or dual-tasking [64]. Cognitive and motor decline co-occur in cardiac patients, both tracking frailty [65].

#### 2.1.5 Cerebellar, basal ganglia, and spinal cord vulnerability

Patients with cardiovascular disease frequently report difficulties with gait variability, balance, and complex motor tasks [18], [28], [66], suggesting dysfunction of cerebellum and basal ganglia in addition to corticospinal pathways. Both structures are among those most vulnerable to ischaemic injury: the hippocampi, corpus striatum, and cerebellar vermi are especially susceptible to hypoxic-ischaemic damage [67], and the latter two provide the sole cortical output of the cerebellum and the principal projection neurons of the basal ganglia respectively. The spinal cord's single midline anterior spinal artery supplies lumbosacral motor neurons that have the highest metabolic demands of any in the cord [68]; whether chronic systemic hypoperfusion produces subclinical injury at this level is not known but is consistent with the disproportionate lower-limb weakness observed in heart failure. When malperfusion is resolved through timely surgical or endovascular restoration of flow, neurological recovery occurs in 58% of spinal cord injuries and 79–84% of stroke and coma presentations [19], [69].

In heart failure, basilar artery inflow is reduced even when global cerebral blood flow appears preserved, indicating selective vulnerability of cerebellum and brainstem [70]. Subclavian steal diverts blood from the posterior circulation during ipsilateral arm exercise, producing transient cerebellar and brainstem ischaemia, while vertebral artery stenosis acts continuously and causes approximately 20% of posterior-circulation ischaemic strokes, with symptomatic patients presenting vertigo, ataxia, dysarthria, and nystagmus [71].

Chronic cerebral hypoperfusion also operates at a microvascular scale. Cerebral small vessel disease is a diffuse arteriolar pathology that accumulates with age and hypertension, producing white matter hyperintensities, lacunar infarcts, and microbleeds across subcortical motor circuits, and impairs gait through both direct damage to frontal-basal ganglia-white matter pathways and indirect loss of the executive control supporting dual-task walking [72]. Meta-analysis links white matter hyperintensity volume to slower gait speed (*r* = −0.23, 95% CI: −0.33 to −0.14) and future falls [73]. Outside the context of stroke, lower cerebral perfusion is associated with clinically meaningful slower gait in a community cohort of 479 adults aged 31–94: at systolic blood pressure of 120 mmHg, each standard deviation lower perfusion produced progressively greater gait slowing with age (0.04 m/s at 65, 0.09 m/s at 85) [74].

Gait impairment in heart failure (slower speed, shorter stride, greater variability) predicts mortality: each 50 m decrease in six-minute walk distance is associated with a 14% higher mortality risk, and distances below 350 m with up to 2.4-fold higher mortality [18]. Heart failure and cardiac arrhythmia are explicit risk factors for subcortical ischaemic vascular dementia, characterised by short-stepped gait and disrupted frontal-subcortical circuits [66]. The increased gait variability and impaired timing are consistent with basal ganglia dysfunction [75]: pure peripheral weakness produces a slow but regular gait, whereas central coordination deficits produce slow and irregular gait.

#### 2.1.6 Reversibility after flow restoration

Cerebral blood flow normalises from 35 ± 3 to 50 ± 3 mL/100 g/min within one month of cardiac transplantation [39], and cognition and exercise capacity also improve, consistent with a causal relationship between perfusion and function. Cognitive recovery likely reflects restoration of prefrontal and associative cortex perfusion; whether the motor cortex specifically benefits has not been tested.

After carotid endarterectomy, the brain's ability to dilate its vessels on demand recovers and a marker of neuronal health in the basal ganglia rises by about a third (*p* = 0.03) [76]; carotid revascularisation broadly improves cognitive and motor performance in neurologically asymptomatic patients [25], [77].

### 2.2 Peripheral hypoperfusion and motor output

When arterial blood flow to peripheral tissues falls below metabolic demand, a predictable sequence of pathological changes arises at both the nerve and muscle levels. Oxygen delivery declines, mitochondrial respiratory-chain function deteriorates as organelles sustain oxidative damage from repeated ischaemia-reperfusion cycles [78], [79], capillary density is roughly 18% lower than in controls [80], and muscle fibres shift from fatigue-resistant oxidative to glycolytic type, atrophy, and eventually undergo necrosis if ischaemia is severe and prolonged [6]. Peripheral nerves affected by the same limb hypoperfusion undergo axonal degeneration [81], producing denervation that compounds the muscle-level deficit [27], [82]. The magnitude of the resulting skeletal-muscle deficits across heart failure, peripheral arterial disease, subclavian stenosis, and aortic stenosis, measured against healthy controls or a pre-procedure baseline, is summarised in Fig. 3.

#### 2.2.1 Peripheral nerve ischaemia and denervation

Peripheral nerves are metabolically active structures that depend on their own blood supply (the vasa nervorum) for axonal transport, Schwann cell function, and impulse conduction. When arterial flow to a limb is compromised, the vasa nervorum are compromised in parallel, and the nerve itself becomes ischaemic. The consequences are demyelination, axonal degeneration, and ultimately motor denervation of the muscle fibres the nerve supplies [81], [83].

The evidence is strongest in peripheral arterial disease. Compound muscle action potential amplitudes are about 30% lower in intermittent claudication and 72% lower in rest pain than in controls (*p* < 0.001), with the stepwise claudication-to-rest-pain fall (*p* = 0.005) showing a dose-response between ischaemia severity and nerve damage; strict axonal criteria are met in roughly a third of nerves tested [27], and 88% of moderately severe PAD patients show progressive multifocal motor neuropathy on follow-up [82]. Muscle biopsies show angular and grouped fibres, indicating active denervation and collateral reinnervation.

In critical limb-threatening ischaemia, the neuropathic injury is more severe [27] and may become irreversible [81]. Ischaemic neuropathy in severely affected limbs can produce sensory loss, motor weakness, and absent reflexes [27] that clinically resemble the presentation of diabetic neuropathy.

Because advanced ischaemic neuropathy is largely irreversible [81], guideline-recommended medical therapy (risk factor control and antithrombotic treatment) and supervised exercise at earlier PAD stages offer the main opportunity to slow PAD progression [32].

#### 2.2.2 Systemic hypoperfusion: heart failure (the failing pump)

In heart failure, skeletal muscle biopsies show mitochondrial dysfunction, capillary rarefaction (an 18% reduction in capillary density, with peak oxygen consumption inversely correlating with rarefaction [80]), and fibre-type shifting in both reduced and preserved ejection fraction phenotypes, though the perfusion deficit is systemic rather than regional [2], [31]. Even during single-leg knee-extensor or handgrip exercise, in which the active muscle mass is too small for cardiac output to be the limiting factor, heart failure patients show approximately 30% lower limb blood flow than controls across both phenotypes, indicating an intrinsic peripheral vasodilatory deficit [30], [84].

Muscle wasting in heart failure overlaps with cardiac cachexia (≈15% of patients [13]), distinguished from sarcopenia by whole-body weight loss driven by systemic inflammation and caloric imbalance [85]. Sarcopenia is amplified by a pathological ergoreflex: muscle afferents drive sympathetic output and peripheral vasoconstriction, starving the working muscle of blood [86]. In healthy controls cardiac output rises by up to 1.1 L/min at peak exercise to support this reflex, whereas in heart failure this response is absent and peripheral vasoconstriction becomes the sole delivery mechanism (systemic vascular conductance falls by up to 13 mL/min/mmHg [87]); chronic sympathetic activation [88] forces the heart to pump against higher resistance, worsening exercise systolic dysfunction [87] and reinforcing the cycle of inactivity and muscle loss [89].

#### 2.2.3 Regional hypoperfusion: peripheral arterial disease (the failing supply chain)

The evidence for this cascade is strongest in peripheral arterial disease, where direct tissue sampling has been extensively performed. PAD gastrocnemius shows selective mitochondrial electron-transport chain deficits [78]. At the fibre level, up to 17% of muscle fibres show central cavities lacking intermyofibrillar mitochondrial activity, and cavity frequency correlates with type I fibre proportion, suggesting oxidative fibres are preferentially affected [79]. The functional cost is substantial: in claudicating patients, peak oxygen consumption is approximately 35% lower than in age-matched controls (16.9 ± 4.4 vs 26.1 ± 4.1 mL·kg^−1^·min^−1^), and can walk on a treadmill for only about half as long before claudication pain stops them [90]. This selective pattern of mitochondrial injury distinguishes ischaemic injury from disuse atrophy, which would produce symmetric weakness without these changes.

Angiogenic and regenerative capacity is compromised. In critical limb-threatening ischaemia, satellite cells differentiate prematurely instead of multiplying, while pro-inflammatory macrophages (twice as abundant as in the same patient's non-ischaemic muscle) withhold the IGF-1 signals satellite cells need [91]. The compensatory angiogenic response to chronic hypoxia is insufficient to meet metabolic demand, reflecting endothelial dysfunction and impaired signalling cascades [92], [93]. Any modest capillary growth cannot keep pace with ongoing damage from hypoperfusion and failed regeneration.

#### 2.2.4 Afferent feedback and central fatigue

Underperfused, metabolically stressed muscle sends amplified afferent signals that suppress central motor drive, creating a remote central consequence of peripheral hypoperfusion. At the same absolute exercise intensity, HFpEF patients with ∼30% lower femoral blood flow and ∼ 35% lower leg vascular conductance develop greater peripheral and central fatigue than controls, with the rate of fatigue acceleration correlating with the slowed blood flow response [30]. At the same relative intensity, fatigue is comparable between groups, indicating that the accelerated fatigue reflects the perfusion deficit rather than an intrinsic neural impairment. The mechanism operates through group III and IV muscle afferents, which sense metabolite accumulation in underperfused tissue and reflexively suppress central motor output [86]. Restoring flow should act on the same loop from the supply side: the delayed rise in peak oxygen consumption after ablation for atrial fibrillation [59] is consistent with a gradual easing of this metabolite signal, though afferent function was not measured.

That perfusion restriction per se is sufficient to drive these neuromuscular changes is shown experimentally: acute cuff occlusion increases motor unit firing rates within seconds to minutes [94], [95], and chronic blood-flow-restriction training shifts recruitment toward higher-threshold fibres [96]. A plausible chronic analogue is the type I to type II fibre-type shift documented in heart failure muscle biopsies [6], [80], consistent with a sustained bias toward higher-threshold motor unit use when perfusion is chronically inadequate.

#### 2.2.5 Reversibility after flow restoration

**Fig. 4:**
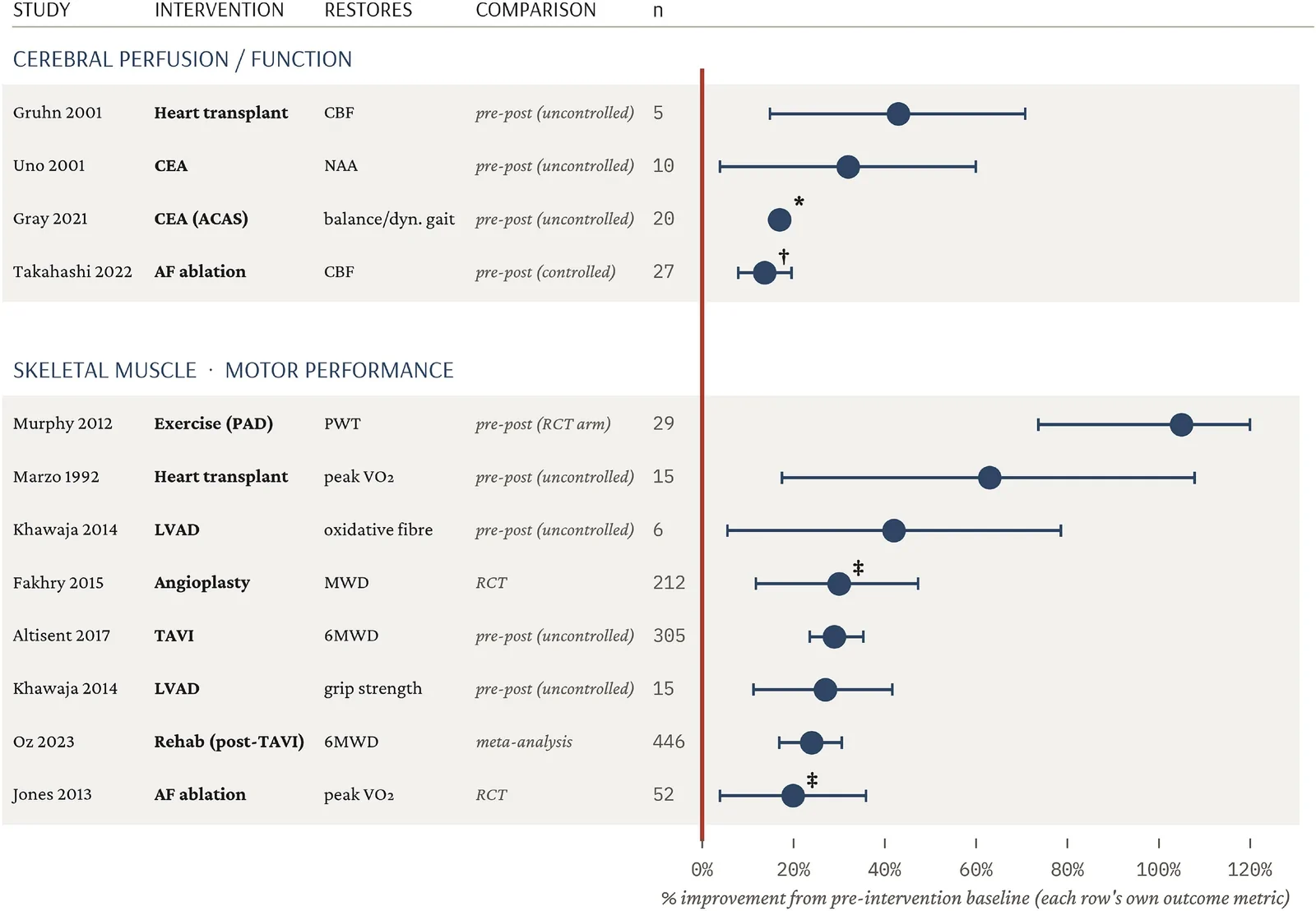
Reversibility of motor-axis deficits after cardiovascular interventions. Forest plot of paired pre-versus-post-intervention improvements across the cardiovascular-motor axis. Each marker is the % improvement reported by the cited paper relative to the pre-intervention baseline; whiskers show 95% confidence intervals unless flagged. The zero line is pre-intervention; dots to the right indicate restoration of function or perfusion. The comparison column gives the design of the plotted comparison, as in Fig. 2, with RCT reserved for rows whose plotted point is the randomised between-arm contrast. n is the number of patients contributing to that estimate, which for several rows is smaller than the parent cohort; for meta-analysis rows it is the number of contributing studies. * improvement magnitude estimated from a published figure or *p*-value bound rather than tabulated mean values, with no numerical confidence interval available. † Takahashi: nonparoxysmal-AF subgroup with restored sinus rhythm (*n* = 27); paired pre/post change, confidence interval derived from the reported *p*-value bound. ‡ between-arm difference at follow-up rather than a within-patient pre/post change: Fakhry, angioplasty + exercise vs exercise alone; Jones 2013 (ARC-HF), catheter ablation vs rate control. Cross-row magnitudes are not strictly equivalent because they compare different outcome types; the % axis unifies magnitudes visually rather than on a single clinical scale. Conditions absent here either lack a published reversibility trial or have no quantitative pre/post motor outcome. ACAS, asymptomatic carotid artery stenosis; AF, atrial fibrillation; CBF, cerebral blood flow; CEA, carotid endarterectomy; HF, heart failure; LVAD, left ventricular assist device; MWD, maximum walking distance; NAA, *N*-acetylaspartate; PAD, peripheral artery disease; PWT, peak walking time; TAVI, transcatheter aortic valve implantation; VO₂, peak oxygen uptake; 6MWD, six-minute walk distance.

Peripheral hypoperfusion-driven pathology is reversible rather than fixed by deconditioning. Left ventricular assist device support raises oxidative fibre proportion (38% to 54%) and skeletal muscle IGF-1 expression roughly tenfold, corrects the growth-hormone/IGF-1 imbalance, and improves grip strength by 26.5 ± 27.5% within 180 days [97]. In PAD, endovascular revascularisation plus supervised exercise improves maximum walking distance by about 30% more than exercise alone (282 m greater at 12 months; *p* = 0.001) [98], though irreversibility thresholds apply once endoneurial fibrosis and satellite-cell depletion have progressed [81], [91]. Recovery magnitudes across these interventions are summarised in Fig. 4.

## 3 Discussion

The evidence reviewed here supports reduced arterial blood delivery as an independent driver of motor impairment, distinct from deconditioning alone. Within-patient asymmetry models hold all systemic variables constant while varying perfusion to a single territory, and the motor deficit consistently tracks the perfusion deficit rather than the shared systemic environment [22], [23], [60]. Dose-response gradients reinforce this pattern: calf muscle area declines progressively with ankle-brachial index even after adjusting for physical activity [9], white matter hyperintensity volume correlates with slower gait speed (*r* = −0.23) [73], and cerebral perfusion predicts gait speed across blood pressure levels [74]. Reversibility after flow restoration, from cerebral blood flow normalising after cardiac transplantation [39] to cognitive and motor function improving after carotid revascularisation [25], [77] and walking distance improving after valve replacement [99], closes the causal loop. This has a practical consequence, developed below. Reduced perfusion appears to be the upstream driver and deconditioning an amplifier that follows from it, but the amplifier does not resolve once flow is restored, so restoring blood flow and rebuilding muscle are likely to be complementary rather than interchangeable.

These findings are distributed along the neuromuscular axis rather than clustered at one level. A patient with heart failure and concurrent PAD may simultaneously show motor cortex thinning ipsilateral to a carotid lesion [23], [60], cerebellar and brainstem hypoperfusion from vertebral stenosis and steal acting on tissues already prone to ischaemic injury [33], [67], [71], accumulating arteriolar injury across subcortical white matter and basal ganglia [72], [73], exposure of spinal motor neurons [68], progressive neuropathy in the ischaemic limb [27], [82], and mitochondrial damage at the fibre level [78], [79]. The motor deficit is the summed output of impairments at every level from cortex to fibre, not the signature of any single lesion. Pure peripheral weakness produces a slow but regular gait, whereas cerebellar and basal ganglia involvement add timing variability and short-stepped irregularity, the pattern identified as subcortical ischaemic vascular dementia and linked explicitly to heart failure and cardiac arrhythmia [66].

The interaction between pathways is not simply additive. When heart failure and atrial fibrillation coexist, the cerebral perfusion deficit is compounded: in atrial fibrillation the rise in middle cerebral artery velocity during exercise is blunted (9% versus 23% in healthy subjects), scaling with the patient's reduced ability to raise cardiac output (r^2^ = 0.55, *p* < 0.01) [54]. Ventricular arrhythmias acutely reduce middle cerebral artery velocity across all groups (heart failure, ischaemic heart disease, and healthy controls), but heart failure patients show impaired cerebral autoregulation during these events: the ratio of cerebral velocity drop to arterial pressure drop during premature ventricular contractions is greater in HFrEF than in controls (*p* = 0.0002), and that impaired-autoregulation ratio is inversely correlated with cognitive performance (*r* = −0.65, *p* < 0.001) [100]. Heart failure with concurrent peripheral arterial disease layers systemic underperfusion onto a territory already compromised by arterial stenosis, worsening outcomes in both phenotypes: concurrent PAD is associated with 31% higher death or hospitalisation in HFrEF [34] and independently increases the risk of death (hazard ratio 1.56) and hospitalisation (hazard ratio 1.36) in HFpEF [35]. Peripheral and central deficits also feed back on each other. Underperfused muscle sends amplified afferent signals that suppress the brain's descending motor commands to the limb, producing measurable central fatigue even when the corticospinal tract is intact [30], [86]. In heart failure, the same reflex drives sympathetic vasoconstriction that increases the resistance the failing heart must pump against [87], so peripheral ischaemia further impairs pump function and the failing pump further reduces perfusion to brain and muscle alike.

The relationship between perfusion and motor output is graded in some territories and threshold-dependent in others. The ankle-brachial index gradient described above is continuous, calf muscle area falling by 13% from the highest to the lowest index (P for trend <0.001) [9]. Cerebral autoregulation, by contrast, maintains cerebral blood flow across a wide range of perfusion pressures but fails below a critical threshold. The clinical presentation reflects the sum of these graded and threshold effects, accumulating at a rate that mirrors the chronicity of the haemodynamic insult.

Valve replacement provides a natural experiment in flow restoration. After TAVI, six-minute walk distance improves by approximately 60 m, though about one-third of patients fail to improve, the gain being smaller with older age, more comorbidities, or peri-procedural bleeding [99]; structured rehabilitation after TAVI adds a further 56 m (236 to 292 m, a 24% increase; *p* < 0.001), even in this uniformly elderly, high-surgical-risk cohort [101]. The pattern is consistent: restoring flow restores function, with the degree of recovery depending on the chronicity of the preceding ischaemia.

Deconditioning alone cannot explain these deficits, for the asymmetry reason given above: a lateralised ischaemic arm or a hypoperfused hemisphere shares its activity level with the unaffected side. On the other hand, deconditioning amplifies every perfusion-dependent pathway through a feed-forward loop: the perfusion deficit reduces motor capacity, reduced motor capacity limits physical activity, and inactivity compounds the deficit through disuse atrophy and further cardiovascular deconditioning. These findings support the hypothesis that intervention should address both the vascular cause and the neuromuscular consequence: endovascular revascularisation (catheter-based angioplasty or stenting of the diseased artery) combined with supervised exercise produces 282 m greater walking improvement than exercise alone [98], while exercise without revascularisation can still outperform stenting alone for treadmill performance [102].

Reduced perfusion is not the only driver of motor impairment here: ageing, chronic inflammation, anaemia, malnutrition, renal dysfunction, diabetes, cardiac cachexia, and frailty each impair neuromuscular function in their own right and frequently coexist with cardiovascular disease [2], [13], [85], [89]. Several act through the same tissue-level biology: the ischaemic PAD limb shows deeper mitochondrial dysfunction, smaller fibres and greater weakness when chronic kidney disease is also present [103], cardiac cachexia adds a catabolic drive through systemic inflammation [85], and the molecular hallmarks of cardiovascular ageing accelerate decline across the vascular, myocardial, valvular, and electrophysiologic domains alike [36], with frailty best read as their integrated endpoint rather than a confounder [36]. Reduced perfusion is therefore one strand among several, and the within-patient asymmetry designs, few as they are, are what separate it from the rest.

These mechanisms span timescales of several orders of magnitude, from beat-to-beat gating of corticospinal excitability [56], [57], to fibre-type and capillary changes over months, to chronic cerebrovascular remodelling over years. The window of recoverability narrows accordingly. When perfusion is restored early, recovery can be substantial, as the mechanical circulatory support data above show [97]. Cardiac transplantation makes the converse point: even when pump function is fully restored, with ejection fraction rising from 16% to 56%, peak oxygen consumption recovers by roughly 63% but remains about 43% below healthy controls (19.2 versus 33.4 mL·kg^−1^·min^−1^), the residual deficit reflecting skeletal-muscle changes that outlast the haemodynamic correction [29]. When intervention is delayed, damage becomes self-sustaining: endoneurial tubes shrink and fibrose to a critical narrowing at approximately four months beyond which reinnervation becomes incomplete [81], satellite cells in critically ischaemic muscle are outcompeted by pro-inflammatory macrophages that block repair [91], chronic cerebral hypoperfusion drives lasting cerebrovascular remodelling that persists even after cardiac output is normalised [104], and the white matter hyperintensities and lacunar infarcts of cerebral small vessel disease persist as structural lesions whose accumulation predicts further gait decline [73]. The case for earlier intervention is therefore strong, before the window of reversibility closes.

Several implications follow, as hypotheses rather than established practice. First, perfusion and neuromuscular status carry different information, so both should be measured: bedside tests of motor function predict mortality and hospitalisation in heart failure [18], and grip strength with lean mass would capture the sarcopenia that goes unrecorded even in cardiovascular trials [105]. Second, treatment may need to pair restoration of flow with resistance-based reconditioning rather than aerobic training alone, because neither works alone: revascularisation with exercise outperforms either by itself [98], [102], training without flow restoration improves function while the tissue continues to denervate [106] and mass gains stay blunted [105], and flow restoration alone leaves a residual deficit after transplantation [29].

Several limitations constrain this framework. It is a synthesis of heterogeneous studies, and the axis is assembled from separate literatures rather than demonstrated in a single dataset: a few links rest on one cohort each, notably the prolonged cortical silent period in carotid stenosis and the reduced basilar inflow in heart failure, much of the evidence is cross-sectional, and no study has measured perfusion and motor unit-level function in the same patients. What the evidence does establish is that the association holds across conditions, tracks the size of the deficit, and reverses when flow is restored, so this is a hypothesis with converging support rather than a settled causal account, and the next step is a study measuring both ends of the axis in the same patients.

## 4 Conclusion

Cardiovascular conditions that reduce arterial blood delivery may impair motor function through multiple converging pathways spanning the full length of the motor axis: cerebral hypoperfusion compromises motor cortical structure and higher-order motor planning; cerebellar and basal ganglia vulnerability produces coordination and gait deficits; spinal motor neurons face ischaemic risk from a weak blood supply; peripheral nerve ischaemia causes progressive denervation; skeletal muscle sustains mitochondrial damage, capillary rarefaction, and regenerative failure; and cardiac-phase signalling gates corticospinal excitability independently of perfusion. Causality is supported by within-patient asymmetry models and by convergent reversibility evidence across transplantation, valve replacement, endarterectomy, and revascularisation.

These mechanisms are further amplified by a feed-forward deconditioning loop, and the case for pairing flow restoration with muscle reconditioning is supported by trial evidence from both revascularisation and exercise interventions.

The critical next step is to conduct studies that simultaneously measure perfusion and motor unit-level neuromuscular function in the same patients. Elective interventions such as TAVI offer a natural experiment: serial assessment before and after flow restoration could isolate each pathway's contribution by its distinct recovery timescale, from immediate normalisation of cardiac-phase motor gating to months-long skeletal muscle reconditioning.

## Declaration of generative AI and AI-assisted technologies in the manuscript preparation process

During the preparation of this work, the authors used Claude (Anthropic, San Francisco, CA, USA) to assist with manuscript prose and to generate Python (matplotlib) scripts that render the figures. No generative AI was used to create or alter image content; all figures were rendered from author-verified numerical inputs. After using this tool, the authors reviewed and edited the content as needed and take full responsibility for the content of the published article.

## CRediT authorship contribution statement

**Matteo Scorcelletti:** Writing – review & editing, Writing – original draft, Visualization, Software, Methodology, Investigation, Formal analysis, Conceptualization. **Armin Weers:** Writing – review & editing, Investigation, Conceptualization. **Bastian Schrader:** Writing – review & editing, Supervision, Conceptualization. **Joachim Schrader:** Writing – review & editing, Supervision. **Alessandro Del Vecchio:** Writing – review & editing, Supervision, Conceptualization. **Albrecht Elsässer:** Writing – review & editing, Supervision, Conceptualization.

## Funding

This research received no specific grant from any funding agency in the public, commercial, or not-for-profit sectors.

## Declaration of competing interest

The authors declare that they have no known competing financial interests or personal relationships that could have appeared to influence the work reported in this paper.
